# Musculoskeletal disorders among dental assistants: a cross-sectional study

**DOI:** 10.1186/s12891-024-07178-7

**Published:** 2024-01-13

**Authors:** Mohammad Aljanakh

**Affiliations:** Department of Restorative Dentistry, College of Dentistry, University of Ha’il, Ha’il, 55473 Saudi Arabia

**Keywords:** MSDs, Musculoskeletal disorders, Dental ergonomics, Dental assistants, Saudi

## Abstract

**Background:**

This study aims to investigate the prevalence of musculoskeletal disorders (MSDs) and associated sociodemographic and work-related factors among dental assistants (DAs) in the hail province, Saudi Arabia.

**Methods:**

Participants were 119 DAs with an average age of 28.9 years (SD ± 4.8 years), of whom 86.6% were females. A self-administered questionnaire which included Nordic Musculoskeletal questionnaire and questions on socio-demographic and work-related factors was used. Descriptive statistics were used to calculate the prevalence of MSDs during the past 12 months and 7 days. Multivariate binary logistic regression statistical tests were used to calculate the association between MSDs and socio-demographic and work-related factors.

**Results:**

The overall prevalence of MSDs among DAs was significantly high, with 85.7% reporting symptoms during the past 12 months and 47.9% during the past 7 days. The shoulders, followed by the lower back, were the most common complaints among participants in the past 12 months and 7 days, followed by the upper back and neck. Multivariate binary logistic regression analysis results show significant associations between MSDs and age, Body-Mass-Index (BMI), physical demands during working hours, work environment and posture awareness, and years of experience.

**Conclusions:**

The prevalence of MSDs among DAs is high, and sociodemographic and work-related factors play an important role in exacerbation of MSDs in DAs.

**Supplementary Information:**

The online version contains supplementary material available at 10.1186/s12891-024-07178-7.

## Background

Work-related Musculoskeletal disorders (MSDs) are common disorders that affect workers across all professions [1]. They also have significant socio-economic consequences. These disorders often result from professional activities and are one of the key concerns of human resource management services and workplace health and safety services. These concerns are due to the intangible consequences of MSDs at the personal and societal levels [2–4]. MSDs are the main cause of disability globally and they frequently cause an incapacity to work, absenteeism, lower job quality, and lower job satisfaction [2, 5]. While Work-related MSDs are common and have a wide range of presentations and causes, MSDs can also be non-work related. Temporomandibular joint dysfunctions (TMDs) are common non-work-related MSDs that involve multifactorial dysfunction of the temporomandibular joint [6]. TMDs have a range of presentations and impacts [7]. A recent study highlighted a connection between TMDs and contact sports in athletes [8]. Additionally, TMDs can affect the quality of life and may even be life-threatening, with a link to suicidal behavior in young people [9].

MSDs are a major concern in dental practice, as significant occupational health issues can occur due to work-related physical demands [10, 11]. Studies have shown that dental professionals have a higher prevalence of MSDs than other healthcare professionals [10–13]. Several risk factors that affect dental professionals’ physical health have been identified, including the use of vibrating devices, the physical demand of work positions, a large number of patients requiring treatment, and a substantial amount of administrative work [14]. The dental practice involves specific postures and repetitive movements in a limited workspace, which pose a considerable risk for MSDs [11, 12, 15].

Internationally, several studies have investigated MSDs among dental assistants (DAs); most of these studies targeted dental professionals as a whole with only a few solely targeting DAs. In Germany, Ohlendorf et al. [16] reported a high prevalence of MSDs among female German DAs, and in another survey of dental professionals in Germany, Holzgreve et al. [4] reported that DAs have a higher risk of developing MSDs than dentists. Partido et al. [17] found that both dentists and DAs demonstrated an inappropriate ergonomic position of body parts during work in the dental chair. In an earlier study, Murtoma et al. [18] reported that 38% of dentists and 12% of DAs have diseases or discomforts related to the profession. In Saudi Arabia, Bakhsh et al. [19] investigated the incidence of MSDs among dentists and allied dental professionals and reported a high incidence of MSDs involving most body parts among DAs. Alghadir et al. [20], investigated the prevalence of MSDs among dental professionals in Saudi Arabia; and found that the most affected body regions were the lower back, shoulders, and neck. Nevertheless, only six of the respondents were DAs.

In this study, we aimed to investigate the prevalence of work-related MSDs in different body parts among DAs in both private and government dental clinics in Ha’il province, Saudi Arabia in the past 12 months and 7 days. We also aimed to determine the associated sociodemographic, occupational, and ergonomic factors associated with MSDs among DAs. We hypothesized that the prevalence of MSDs among DAs will be high and that work-related characteristics will have an impact on the prevalence of MSDs. In addition, this study’s findings can help decision-makers in dental teams gain new insights into the factors that may affect DAs profession.

## Methods

### Study design and sample

This study is a self-administered, questionnaire-based, cross-sectional study aiming to assess the prevalence of MSDs as well as the impact of associated sociodemographic, occupational, and ergonomic factors among DAs in Ha’il province, Saudi Arabia. This study spanned six months starting in May 2022. Due to the lack of a complete list of all DAs, randomization was not possible, and a non-probability sampling method was used to recruit DAs. Roscoe’s rule of non-probability adequate sampling was followed [21]. The estimated number of DAs in Ha’il province was calculated based on the number of dentists and dental clinics, which was approximately 200 subjects.

### Participants

All licensed government and private DAs who practice full-time in Ha’il province and are over 18 years old with at least 6 months of practice experience were eligible to participate in this study. However, DAs with a history of musculoskeletal trauma, systemic disorders, pregnancy, and active-resistant exercise trainees were excluded from this study. Participants who refrained from answering the questionnaire were also excluded.

### Variables

The outcome variables were the presence or absence of complaints (no/yes) of MSDs in the neck, elbows, shoulders, wrists/hands, upper back, hips/thighs, lower back, knees, and ankles/feet. The predictor variables were sociodemographic, occupational characteristics, and ergonomic knowledge.

The variables were categorized as categorical or continuous variables. The categorical variables were gender (male/female), setting of work (government/private dental clinic), work environment and posture awareness (no/yes), and MSDs in each body part (no/yes). The continuous variables were age (years), body mass index (BMI) (kg/m2), years of experience (scale from 0 to 4), work hours per week (scale from 0 to 3), work-related physical demands (scale from 0 to 4) and following ergonomic work positions (scale from 0 to 3).

### Questionnaire

The data collection tool in this study was based on a self-administered Nordic questionnaire [22]. The questionnaire in this study is a validated Arabic-English version that has been previously used to assess work-related MSDs and related factors in similar populations [19, 23, 24]. The questionnaire consisted of three parts preceded by five questions related to the inclusion criteria (Supplementary Material 1).

The first part contains four sociodemographic questions about gender, age, weight, and height. The second part of the questionnaire includes six occupational characteristics and ergonomic knowledge questions about the setting of work, years of experience, work hours per week, work-related physical demands, work environment and posture awareness, and following ergonomic work positions. The third part included a Nordic questionnaire on MSDs of the neck, elbows, shoulders, wrists/hands, upper back, hips/thighs, lower back, knees, and ankles/feet [22]. This part measures the work-related MSDs of participants during the past 12 months and 7 days.

To minimize bias in this study the administrations of both government and private clinics were contacted to obtain permission for their DAs to participate in this study. To minimize the recall bias, a standardized questionnaire with clear instructions was used.

The paper copies of self-administered questionnaires were distributed to both government and private DAs. The questionnaires for government DAs were distributed and collected by the personnel of Dental Administration at the Ministry of Health in Ha’il province, while the questionnaires for private DAs were distributed and collected by the researcher. Two weekly reminders were sent to the clinic’s administrations for completing the questionnaires.

### Data and statistics

The statistical analysis of this study used IBM® SPSS® Statistics software, version 24. Descriptive statistics of socio-demographics, work characteristics, ergonomic knowledge, and prevalence of MSDs were summarized using means, standard deviation (SD), percentages, and frequencies. Binary logistic regressions were used to detect the associations between the MSDs in different body parts in the past 12 months as outcome variables and the socio-demographics, work characteristics, and ergonomic knowledge as predictor variables. The statistical significance was set at a P*-value* level of 0.05 and the odds ratios (ORs) were calculated with 95% confidence intervals (CIs) to estimate the association. Before conducting binary logistic analysis, the fit of the model was tested and confirmed. Any possible confounding effects of predictor variables were controlled by including all predictor variables in the model. The results were summarized and presented in tables and a graph.

### Ethical considerations

This study was performed in accordance with the Helsinki Declaration and was approved by the University of Ha’il ethics committee, reference number of H-2022-L76. Participation in this study was anonymous and voluntary, and informed consent was required before answering the questionnaire. The reporting of this study conforms to the recommendations and checklist items outlined in the STROBE statement for observational studies [25].

## Results

### Sociodemographic

The questionnaire was completed by 134 DAs, with a response rate of 67%. However, 119 participants were part of this study after 15 DAs were excluded because they did not meet the required inclusion criteria. Only 16 (13.4%) out of 119 participants were males, whereas 103 (86.6%) were females. The mean age of the participants was 28.9 ± 4.8 years. Out of a total of 119 DAs, 76 (63.9%) were practicing in private clinics, while the remaining 43 (36.1%) were practicing in the government clinics. A total of 76 (63.9%) participants worked in private dental-based clinics, while 43 (36.1%) worked in government dental clinics. The average height of participants was 163.8 ± 9.1 cm, and the average weight was 67.9 ± 16 kg. According to the BMI, 52 of the participants (43.7%) were overweight and 55 (46.2%) had a normal BMI, eight were obese (6.7%), and only four (3.4%) were underweight. Table 1 presents sociodemographic data.

**Table 1:** Socio-demographic data of dental assistants, (*n* = 119)

	n (%) or mean ± SD
**Gender**	
Female	103 (86.6%)
Male	16 (13.4%)
**Age (Years)**	28.9 ± 4.8
**Setting of work**	
Government	43 (36.1%)
Private dental clinic	76 (63.9%)
**Height (cm)**	163.8 ± 9.1
**Weight (kg)**	67.9 ± 16
**BMI**	
Underweight (< 18.5 kg/m2)	4 (3.4%)
Normal (18.5–25 kg/m2)	55 (46.2%)
Overweight (25-29.9 kg/m2)	52 (43.7%)
Obese (> 30 kg/m2)	8 (6.7%)

### Work characteristics

About half of the participants had less than ten years of practice as a dental assistant - while the other half had over ten years of experience. For work hours per week, 46 (38.7%) of the participants used to work over 45 h per week, while 42 (35.3%) worked 40 to 45 h per week. For physical demands during work hours, 46 (38.7%) of the participants reported having a daily physical demand 50–75%, followed by 38 (31.9%) who had 30 to 50%, while 30 (25.2%) had over 75% daily physical demand. Table 2 presents the work characteristics of DAs.

**Table 2:** Work characteristics of dental assistants, (*n* = 119)

Work characteristic	n (%)
**Years of experience (years)**	
0.5-5 years	38 (31.9%)
5–10 years	26 (21.8%)
10–15 years	46 (38.7%)
15–20 years	6 (5%)
Over 20 years	3 (2.5%)
**Work hours per week**	
Under 35 h	6 (5%)
35–40 h	25 (21%)
40–45 h	42 (35.3%)
Over 45 h	46 (38.7%)
**Work-related physical demands**	
Under 30%	3 (2.5%)
30–50%	38 (31.9%)
50–75%	46 (38.7%)
Over 75%	30 (25.2%)
100%	2 (1.7%)

### Ergonomic knowledge of the dental assistants

Regarding work environment and posture awareness, 72 (60.5%) of the participants indicated that they do not have work environment and posture awareness during work, while 47 (39.5%) indicated that they were aware of the proper work environment and posture in their clinical work. Regarding following ergonomic working positions, 19 (16%) of participants reported that they never followed ergonomic working positions, while 13 (10.9%) of the participants reported that they rarely did. On the contrary, 40 (33.6%) indicated that they follow ergonomic working positions most of the time, while 47 (39.5%) do so all the time. Table 3 presents the ergonomic knowledge of the DAs.

**Table 3:** Ergonomic knowledge of the dental assistants, (*n* = 119)

**Work environment and posture awareness**	n (%)
No	72 (60.5%)
Yes	47 (39.5%)
**Follow ergonomic working positions**	
Never	19 (16%)
Rarely	13 (10.9%)
Most of the time	40 (33.6%)
All the time	47 (39.5%)

### Prevalence of MSDs

**Fig. 1:**
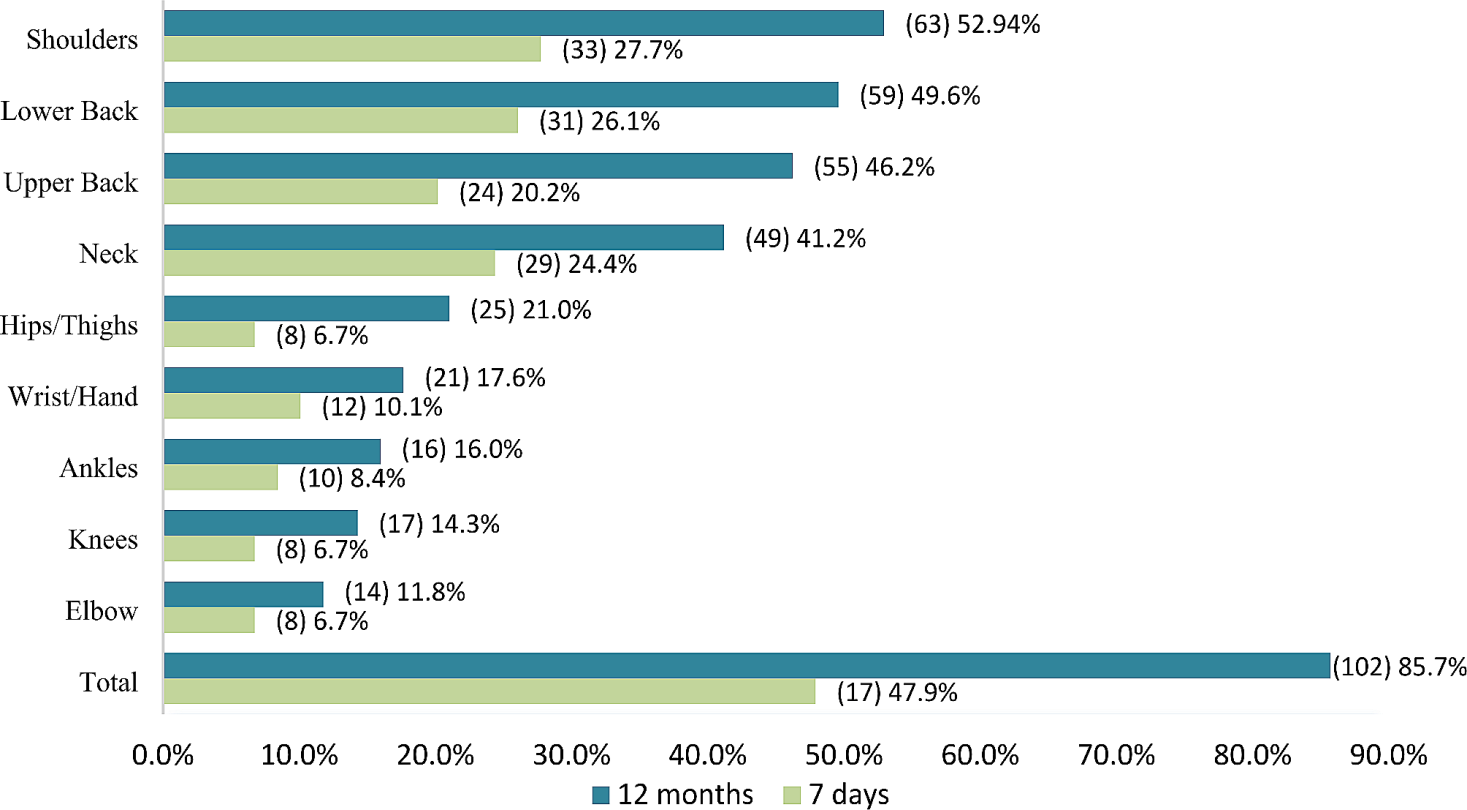
The frequencies and percentages of Musculoskeletal Disorders in different body regions in the past 12 months and 7 days of studied dental assistants, (*n* = 119)

A total of 102 (85.7%) respondents indicated that they had MSDs at least in one or more body regions within the past 12 months, while 57 (47.9%) indicated they had pain in the past 7 days in at least one or more body regions. Shoulders followed by lower back were the most common complaints among participants, with approximately two-thirds reporting shoulders and lower back pain within the past 12 months and about one-third within the past 7 days in these body regions. The next most common complaint was the upper back and neck, with nearly half reporting pain within the last 12 months and a-fourth reporting pain within the past 7 days in these body regions. In contrast, participants reported less frequent pain in the hips, ankles, knees, and elbows in these body regions. The frequencies and percentages of MSDs in the past 12 months and 7 days are shown in Fig. 1.

### Association between MSDs and potential influencing predictors

Table 4 shows the results of the multivariable binary logistic regression analysis measuring significant predictors of sociodemographic, work characteristics, and ergonomic knowledge and the occurrence of MSDs among DAs in different body regions at 12 months. For the significant predictors, we found that age, BMI, physical demands during working hours, work environment and posture awareness, and years of experience were significantly associated with the different body regions affected by MSDs.

As age increased, the odds of MSDs were significantly higher in the elbows (OR: 1.1; 95%CI: 1.0–1.3) and the upper back (OR: 1.2; 95%CI: 1.0–1.5). Likewise, with higher BMI, the odds were higher of MSDs in ankles (OR: 3.1; 95%CI: 1.0–9.0), knees (OR: 3.9; 95%CI: 1.0–15.2), and hip (OR: 3.2; 95%CI: 1.1–9.2).

Physical demands during working hours were found to be a significant predictor for musculoskeletal disorders in the hip (OR: 3.5; 95%CI: 1.3–8.9), lower back (OR: 2.5; 95%CI: 1.3–4.9), and upper back (OR: 4.1; 95%CI: 1.6–10.6). Work environment and posture awareness were found to be significant predictors for musculoskeletal disorders in the shoulder (OR: 0.14; 95%CI: 0.037–0.53), indicating that individuals who have work environment and posture awareness are 86% less likely to develop MSDs in the shoulder compared to those who do not. Years of experience were found to be a significant predictor for musculoskeletal disorders in the knees (OR: 2.9; 95%CI: 1.0–8.5).

**Table 4:** Multivariate binary logistic regression analysis of Significant predictors associated with the occurrence of musculoskeletal disorders at 12 months, (*n* = 119)

Predictor variable	Body Part	OR (95% CI)
Age	Elbows	1.1 (1.0, 1.3)
	Upper back	1.2 (1.0, 1.5)
BMI (body mass index)	Ankles	3.1 (1.0, 9.0)
	Knees	3.9 (1.0, 15.2)
	Hip	3.2 (1.1, 9.2)
Physical demands during working hours	Hip	3.5 (1.3, 8.9)
	Lower back	2.5 (1.3, 4.9)
	Upper back	4.1 (1.6, 10.6)
Work environment and posture awareness (No)	Shoulder	0.14 (0.03, 0.5)
Years of experience	Knees	2.9 (1.0, 8.5)

Predictor variables included in the model: gender, age, BMI, work setting, years of experience, working hours per week, physical demands during working hours, work environment and posture awareness, follow ergonomic work positions.

## Discussion

This study aimed to investigate the prevalence of MSDs in a group of DAs in Ha’il province, Saudi Arabia, and to determine the associated sociodemographic, occupational, and ergonomic factors. Only a few previous studies have included DAs; most of these studies have included dentists or dental practitioners in their studies [20, 26]. Therefore, this study’s importance stems from the fact that it focuses on the work conditions of DAs. In addition, this study forecasts the relationship between MSDs and socio-demographics, and work-related associated factors.

The results of this study showed that the general MSDs prevalence in all body parts among DAs is significantly high in the past 12 months (85.7%) and 7 days (47.9%). These results are in line with previous studies in different countries that reported a high MSDs prevalence among DAs in all body parts [16, 19, 27]. Akesson et al. [27] in Sweden reported an MSDs prevalence of 88% in the past 12 months and 62% in the past 7 days among DAs. Ohlendorf et al. [16] in Germany reported that the MSDs prevalence among DAs was 97.5%.

Our results for the individual body regions showed that the most affected body regions in the past 12 months and 7 days were the shoulders (52.9%), lower back (49.6%), upper back (46.2%), and neck (41.2%). These findings are consistent with systematic review and meta-analysis results by Lietz et al. [14], which reported that the neck, lower back, shoulder, and upper back were the most commonly affected body regions among dental professionals. However, contrary to other studies, our study found a lower prevalence of MSDs in these individual body regions, which might be due to the younger average age in our sample (28.9 ± 4.8). For instance, Bakhsh et al. [19] reported higher levels of complaints in the shoulders (61.4%), lower back (62.6%), neck (63%), and upper back (52.6%) among DAs in Saudi Arabia in the past 12 months. Similarly, Ohlendorf et al. [16] reported higher complaints among qualified DAs in Germany, with 70.2% of complaints of shoulders, 60.1% of lower back complaints, 48% of upper back complaints, and 85% of neck complaints in the past 12 months. Additionally, in Sweden, Akesson et al. [27] reported that 62% of DAs experienced symptoms in both shoulder and neck in the past 12 months.

The complaints in the past 12 months in hips/thighs, wrist/hand, ankle/foot, knees, and elbows were less prevalent in our study (reported at 21%, 17.6%, 16%, 14.3%, and 11.8%, respectively) compared to other studies. However, such complaints were much higher in other studies. In Saudi Arabia, Bakhsh et al. [19] reported a higher prevalence of pain in the hips/thighs 36.1%, wrist/hand (52.2%), ankle/foot (48.6%), and 42.6% in the knees among DAs in the past 12 months. Similarly, Ohlendorf et al. [16] reported, a higher prevalence of wrists/hands pain (31.8%) and a comparable prevalence of pain in hips/thighs (15%), ankle/foot (13.1%), and knees (16.3%). Moreover, Akesson et al. [27] in Sweden reported a higher percentage of pain involving wrists/hands (27%) and comparable results for elbows (15%).

Dentists and DAs operate within a shared work environment, yet they exhibit varying manifestations of MSDs. Most previous studies showed a higher overall prevalence of MSDs in DAs compared to dentists. For instance, a study conducted in Ha’il province, Saudi Arabia found a lower prevalence of total MSDs among dentists (77.9%) compared to the total of MSDs among DAs in this study (85.7%) [23]. Holzgreve et al. [4] similarly reported significantly higher MSDs among DAs than among dentists, which was attributed to differences in the occupational demands of the two professions. Previous studies also identified that DAs were more affected in specific body regions compared to other dental health professionals. Radanović et al. [28] found that dental assistants’ neck discomfort was the highest (90.9%) among dental workers in Serbia. Proteau et al. [29] reported that the prevalence of neck pain among female DAs in Canada was the highest among all dental professionals. Bakhsh et al. [19] reported that DAs had significantly higher complaints of MSDs compared to dental technicians. This different presentation of MSDs between DAs and other dental professionals can be explained by the different tasks and postures in dental clinics. These differences were recently investigated in a laboratory study by Ohlendrof et al. [30], which attributed these differences to the different nature of work and tasks between the two professions. Furthermore, there is some evidence that female dental professionals might have higher MSDs than males [16, 20, 31]. However, several studies, including this study, did not find a significant association of gender on MSDs [19, 32, 33].

The results of this study showed that DAs had long work hours per week and high physical demand during work, which may explain their high MSDs prevalence. To manage and prevent MSDs, some approaches are possible. According to a systematic review by Lietz et al. [34], some ergonomic interventions, such as ergonomic chairs and training courses, can help prevent or reduce MSDs among dental professionals. Another approach is to increase DAs staff and spread the workload, which might reduce work hours and physical demands for DAs. However, this might raise costs, and cause disputes or dental team communication challenges. Thus, it is crucial to weigh the pros and cons of hiring additional people and investigate cheaper alternatives. Raising awareness and implementing DAs ergonomics might decrease health concerns, and improve comfort, and productivity.

Our findings on the ergonomic knowledge of the dental assistants indicated a discrepancy between the awareness and practice of ergonomic principles by DAs. While 87% of them reported following ergonomic work positions most or all of the time, 60.5% reported a lack of awareness regarding the work environment and posture. This suggests that many DAs may lack a proper understanding of ergonomics and appropriate positioning, despite their apparent conformance. Possible causes for this disparity include informal or insufficient education and training and limited or inaccurate knowledge of ergonomic work positions. These explanations highlight regions for improvement in this group’s ergonomics education and training.

In this study, we found no significant associations between MSDs and gender, work setting, or working hours per week. However, we did identify significant associations between MSDs and age, BMI, physical demands during working hours, work environment and posture awareness, and years of experience. Our study showed that dental assistants with higher years of experience had a higher level of MSDs. This is consistent with the findings of Nir Uziel et al. [35], who reported that years of professional experience is one of the risk factors that contributed to work stress and burnout among studied dental assistants. However, Bakhsh et al. [19] found that dental professionals with fewer years of experience had lower levels of MSDs. Contrary to our findings, Samat et al. [5] found that age was not a significant risk factor for MSDs. Our study identified a significant association between MSDs and BMI, consistent with the findings of Traversini et al. [3]. In addition, Brady et al. [36] identified being overweight as a risk factor affecting MSDs in young adult women. Previous studies reported gender as a risk factor for MSDs [16, 20], but our results did not show a significant association between MSDs and gender. This result is consistent with the findings of Bakhsh et al. and Hashim et al. [19, 33], who also found no significant association between gender and MSDs.

The unique characteristic of MSDs and their associated risk factors is their varied distribution across different populations, professions, and activities such as sports. The contrast in the prevalence of Musculoskeletal Disorders (MSDs) among health workers, physiotherapists, and athletes reveals the intricate interplay of occupation-specific risk factors in the onset of MSDs.

The high prevalence of MSDs among healthcare workers is well-documented. For instance, Alnaami et al. [13], identified a higher prevalence than the reported in this study of lower back MSDs among health workers, with 73.9% reporting lower back pain in the previous 12 months. Significant risk factors included employment in secondary/tertiary hospitals, obesity, and a history of spinal trauma.

Abdulmoughni et al. [37] found that 40.3% of healthcare workers experienced occupational injuries, primarily MSDs, with lifting tasks and physical exertion as significant risk factors. Jacquier Bret and Gorce [38] conducted a systematic review and discovered that surgeons and dentists had the highest prevalence of lower back pain (over 60%), shoulder and upper extremity disorders (35 to 55% MSDs complaints), while nurses reported the most lower limb complaints (over 25%). The primary cause, maintaining and repeating awkward postures, aligns with our findings, potentially due to the nature of the job healthcare workers undertake.

Studies focusing on hospital staff, such as those by Landry et al. [39] and Jellad et al. [40], reported a lifetime low back pain prevalence of 70.9% and a total MSD prevalence of 65.4% respectively. Similarly, Homaid et al. [41] reported a high prevalence of low back pain among operating room staff. These findings highlight the significant role of occupation-specific risk factors in MSD prevalence.

In contrast to our findings, studies on physical therapists reported a lower overall prevalence of MSDs. Alnaser & Aljadi [42] found a 48% prevalence in the past year among physical therapists, with significant risk factors including conducting manual therapy, lifting, and shifting patients. Khairy et al. [43] reported an 82.6% prevalence over the past two years, with risk factors including older age and more years of experience among Egyptian Physiotherapists. In a systematic review, Gorce and JacquierBret [44] found varying prevalence rates of MSDs among physiotherapists, with the highest being lower back pain (40.1%), followed by thumbs (35.4%). This finding is unique to the physiotherapy profession due to the repetitive use of fingers compared to some other professions. These results contrast with our findings, potentially due to differences in task nature and occupational physical demands.

In agreement with our results, Al Shammari et al. [45] reported a prevalence of 88.9% of MSDs in the past year among radiologists, with risk factors including being female, older age, and reviewing CT scans or ultrasound images and this might be due to some similarities of posture and repetitive movements of DAs with radiobiologists.

Moreover, DAs may be exposed to risk factors for MSDs that differ from those of different sports due to the variable physical demands and risk factors between them. The affected body areas among DAs are different than the corresponding injured body area reported for some sports. For instance, Bromley et al. [46] found that certain body areas were most frequently injured in different combat sports, with the head/face being most injured in boxing and wrestling, the wrist in boxing, and the lower back in boxing and judo. In contrast to our findings, a systematic review by Maselli et al. [47] found a significantly lower prevalence of low back pain in runners (0.7–20.2%) and identified different risk factors than those in our study. For runners, the risk factors for low back pain included having more than 6 years of running experience, a BMI greater than 24, being taller, not performing traditional aerobic activity weekly, having a restricted range of motion of hip flexion, having a difference in leg length, and having poor hamstrings and back flexibility.

In this study, DAs have the highest prevalence of shoulder MSDs which identifies distinct occupational risk factors of DAs. The higher prevalence of shoulder MSDs among DAs (52%) suggests greater exposure to risk factors, such as prolonged overhead work, while the substantially greater prevalence of upper back MSDs (46.2% vs. 17.7%) indicates distinct risk factors related to bending and twisting during dental procedures.

Preventive measures for MSDs among dental professionals are a promising region of research. There is no doubt that preventing or reducing MSDs is the best approach. In a study of the effects of exercise on MSDs, Holzgreve et al. [48], found that a 10-week resistance training program significantly reduced musculoskeletal pain in dentists and DAs and recommended it as a behavioral MSDs preventive measure of MSD for dental professionals. Furthermore, a systematic review by Maselli et al. [47] found that running could be a protective factor against the onset of lower back pain. In another preventive approach, Anshasi et al. [49], reported promising outcomes of using Kotter’s change management model to assist dental professionals manage and prevent MSDs in their work environment.

The limitations of this study include the self-report questionnaire-based nature of the study which may be subject to reporting bias; hence it did not measure MSDs clinically using diagnostic measures. Another limitation is the lack of randomization of the study sample due to the inability to obtain needed data. To improve the well-being and productivity of dental professionals, we recommend dental ergonomics continuous educational sessions and workshops held by employers. Extending knowledge and practice on dental ergonomics in all dental curricula and including ergonomics as part of student assessment is also a priority.

## Conclusions

In summary, the prevalence of MSDs among the studied subgroup of Saudi DAs was high. The shoulder was the most affected body region, followed by the lower back, upper back, and neck. MSDs are significantly associated with several sociodemographic, work characteristics, and ergonomic knowledge risk factors. These findings emphasize the important need for targeted interventions and further research to address the high prevalence and risk factors of MSDs in this population.

### Electronic supplementary material

Below is the link to the electronic supplementary material.

Supplementary Material 1

## Data Availability

The data that support the findings of this study are available from the corresponding author upon reasonable request.

## Abbreviations

MSDs: Musculoskeletal disorders
DAs: Dental assistants
BMI: Body Mass-Index
