# Supplementary material for: Musculoskeletal disorders among dental assistants: a cross-sectional study
Source: BMC Musculoskelet Disord. 2024 Jan 13;25:64. doi: 10.1186/s12891-024-07178-7 (PMC10787391; doi:10.1186/s12891-024-07178-7)
Supplement: Supplementary file 1 — Supplementary Material 1 [file 12891_2024_7178_MOESM1_ESM.docx]

| ID: ………………. | | | | | | | | | |
| --- | --- | --- | --- | --- | --- | --- | --- | --- | --- |
| Q1.1 What is your gender: | | | 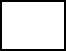 Male | | - female | | | | |
| Q1.2 How old are you: | | | …………. years | | | | | | |
| Q1.3 What is your work setting?  (Please put mark in one box only) | | | - Government dental clinic - Private dental clinic | | | | | | |
| Q1.4 What is your height in centimeter? | | | | …………. cm | | | | | |
| Q1.5 What is your weight in kilograms? | | | | …………. Kg | | | | | |
| **Work characteristics** | | | | | | | | | |
| Q2.1 How many years have you practiced as dental assistant? | | | | | | ………………………….. years | | | |
| Q2.2 How many hours do you work per week on average? | | | | | | | | | …………… hour |
| Q2.3 What percentage of your working hours involves physical activity?  (Please put mark in one box only)   - Less than 30% - 30-50% - 50-75% - More than 75% - 100% | | | | | | | | | |
| **Ergonomic knowledge** | | | | | | | | | |
| Q3.1 Are you Aware of proper work environment and posture? (Please put mark in one box only) | | | | | | | - No - Yes | | |
| Q3.2 How often do you Follow proper ergonomic work positions? (Please put mark in one box only) | | | | | | | - Never - Rarely - Most of the time - All the time | | |
| **Nordic Musculoskeletal Questionnaire (NMQ):** | | | | | | | | | |
| Please indicate if you have experienced pain, discomfort, or numbness in any of the following body regions within **the last 7 days** **AND** within the **last 12 months**. You may mark more than one body region if applicable. **(Mark the appropriate box/boxes)** | | | | | | | | | |
| Body region | | **Last seven days** | | | | | | **Last 12 months** | |
| 1 | Neck | 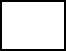 | | | | | | 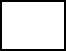 | |
| 2 | Shoulders | 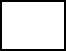 | | | | | | 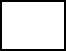 | |
| 3 | Upper Back | 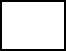 | | | | | | 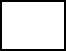 | |
| 4 | Elbow | 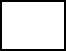 | | | | | | 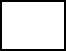 | |
| 5 | Wrist/Hand | 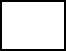 | | | | | | 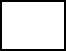 | |
| 6 | Lower Back | 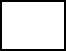 | | | | | | 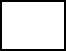 | |
| 7 | Hips/Thighs | 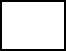 | | | | | | 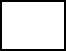 | |
| 8 | Knees | 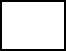 | | | | | | 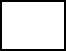 | |
| 9 | Ankles | 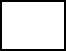 | | | | | | 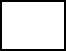 | |
